# Anemia is an independent predictor of increased overall mortality in older adults – results from the PolSenior study

**DOI:** 10.3389/fpubh.2026.1908528

**Published:** 2026-10-28

**Authors:** Arkadiusz Styszyński, Aleksander J. Owczarek, Jerzy Chudek, Małgorzata Mossakowska, Alicja Klich-Rączka, Sławomir Tobis, Aleksandra Kaluźniak-Szymanowska, Beata Kaczmarek, Katarzyna Wieczorowska-Tobis

**Affiliations:** 1 Laboratory for Geriatric Medicine, Department of Palliative Medicine, Poznan University of Medical Sciences, Poznan, Poland; 2 Health Promotion and Obesity Management Unit, Department of Pathophysiology, Faculty of Medical Sciences in Katowice, Medical University of Silesia in Katowice, Katowice, Poland; 3 Department of Internal Medicine and Oncological Chemotherapy, Faculty of Medical Sciences in Katowice, Medical University of Silesia in Katowice, Katowice, Poland; 4 International Institute of Molecular and Cell Biology, Warsaw, Poland; 5 Department of Internal Medicine and Gerontology Collegium Medicum of the Jagiellonian University in Krakow, Kraków, Poland; 6 Department of Occupational Therapy, Poznan University of Medical Sciences, Poznan, Poland

**Keywords:** aging and public health, anemia in older adults, comprehensive geriatric assessment, functional status, mortality

## Abstract

**Background:**

Although anemia in old age is traditionally viewed as a benign bystander of the aging process, its systemic impact on population health remains underestimated. This study evaluates the long-term health consequences of established hemoglobin concentration cut-off values by analyzing their independent impact on 10-year mortality and functional independence among community-dwelling older adults.

**Participants and methods:**

3,806 people aged 65 to 103 (1811 women, 1995 men) participated in the PolSenior project, a nationwide cross-sectional study with a 10-year prospective follow-up for mortality. We used ROC curve analysis to determine sex-specific hemoglobin cut-offs for mortality risk. Comprehensive geriatric assessments, including activities of daily living (ADLs/IADLs), cognitive status, nutritional risk, and inflammatory markers (IL-6, CRP), were analyzed across hemoglobin ranges using multivariable stepwise Cox proportional hazard regression stratified by age and renal function.

**Results:**

During the 10-year follow-up period, 56.9% of the participants died. We established empirical hemoglobin cut-off values above which mortality increases sharply: 13.3 g/dL for women and 13.9 g/dL for men. Subgroups with results below these thresholds exhibited significantly higher mortality (*p* < 0.001) and a higher incidence of dementia, depression, malnutrition, falls, and severe impairment in ADL/IADL. Multivariate analysis confirmed that the increased risk of death associated with low hemoglobin levels persisted independently of the presence of major chronic diseases and systemic inflammation.

**Conclusion:**

This study provides the first nationwide evidence of distinct, sex-specific hemoglobin thresholds that independently influence long-term mortality and declines in functional ability. These findings highlight a key public health challenge: even a slight decrease in hemoglobin concentration represents a significant, modifiable risk factor for the loss of independence among the older adults. Public health strategies for healthy aging should incorporate proactive, community-based screening for anemia to delay the onset of disability and reduce the socioeconomic burden on aging healthcare systems.

## 1 Introduction

The United Nations World Social Report 2023 projects that the number of people aged 65 or older will double from 761 million in 2021 to 1.6 billion by 2050. Those aged 80 or older are increasing even more rapidly (1). This rapid growth highlights the need for effective prevention and treatment of illnesses leading to dependency and death. Healthcare demand relates to the population’s health, especially the older individuals with multiple costly morbidities (2). Addressing common but underrecognized medical issues is essential to prevent dependency among older adults.

For decades, mild anemia in older adults was not considered a major clinical issue, but rather an “innocent bystander” to the aging process (3). Some researchers have even suggested lowering this demographic’s reference values for blood hemoglobin levels (4). However, as the population ages, researchers have focused on the factors behind the decline in functional status and the gradual loss of independence in older individuals; anemia is among these factors. Since the early 2000s, increasing evidence indicates that even mild anemia in older adults can worsen motor and cognitive abilities (5–7). The Health and Anemia (H&A) study demonstrated that mild anemia (defined as a hemoglobin level of at least 10 g/dL) increases the risk of hospitalization and mortality within a three-year follow-up period (8). The higher mortality rates in older adults with mild anemia are partly attributed to poorer survival outcomes in the context of acute illnesses or worsening chronic conditions (9). However, findings from the Established Populations for Epidemiologic Studies of the Elderly (EPESE) indicate that anemia in older adults correlates with an increased risk of hospitalization and death, even in those without clinically significant diseases (10).

Some evidence suggests that low hemoglobin levels within the normal range may be associated with increased mortality in older adults (11). However, to the best of our knowledge, no previous nationwide study has identified a hemoglobin level at which mortality increases in older adults living in the community. Our study aims to fill this gap.

It is debatable whether the higher mortality rates in older adults with low hemoglobin levels occur independently of the underlying causes of anemia or if low hemoglobin is simply a marker for diseases that more directly elevate mortality risks. Studies investigating the clinical effects of unexplained anemia indirectly address this issue. An observational study in Bergen, Norway, found no increased risk of readmission or death in patients with unexplained anemia (12). However, this research focused only on the first year post-acute hospitalization and was limited to a specific group of patients discharged from a long-term care unit (12). In the H&A study, 11- and 15-year survival rates were evaluated by cause of anemia in two groups of older adults: those aged 65 to 80 and those over 80 (13). In the younger cohort, a link was identified between mortality and mild anemia due to chronic kidney disease (CKD), chronic inflammation, deficiency anemia, and unexplained anemia. Conversely, for individuals over 80, this link was only noted for anemia related to chronic inflammation and CKD (13). Data from the National Health and Nutrition Examination Survey (NHANES) were examined to evaluate mortality risk among individuals aged 60 and older who were anemic and frail. This analysis involved 6,406 older adults over a 13-year follow-up period. Compared to non-anemic and frail participants, those who were anemic and frail faced nearly a four-fold greater risk of all-cause mortality, over four times the risk of cardiovascular mortality, and more than five times the risk of non-cardiovascular or cancer-related mortality (14).

Our previous studies assessed the prevalence of anemia and socioeconomic factors among older adults in Poland (15). We characterized the etiology of anemia in this population (16). The current study aims to determine the impact of low hemoglobin on survival and functional outcomes among Polish seniors.

## 2 Participants and methods

### 2.1 Study population

Assessment of anemia was part of the PolSenior project, a nationwide cross-sectional study with a 10-year prospective follow-up for mortality. The study, conducted from 2007 to 2012, aimed to evaluate the medical, psychological, social, and economic aspects of aging in Poland (17). The study sought to define the current status of older subjects, considering various dimensions of aging and the needs of Polish seniors. The analysis of overall survival included 3,806 participants – 1811 women (47.6%) and 1995 men (52.4%) with complete blood count (CBC), not on renal replacement therapy (RRT), without current or past cancer, and with known survival time (Figure 1).

**Figure 1:**
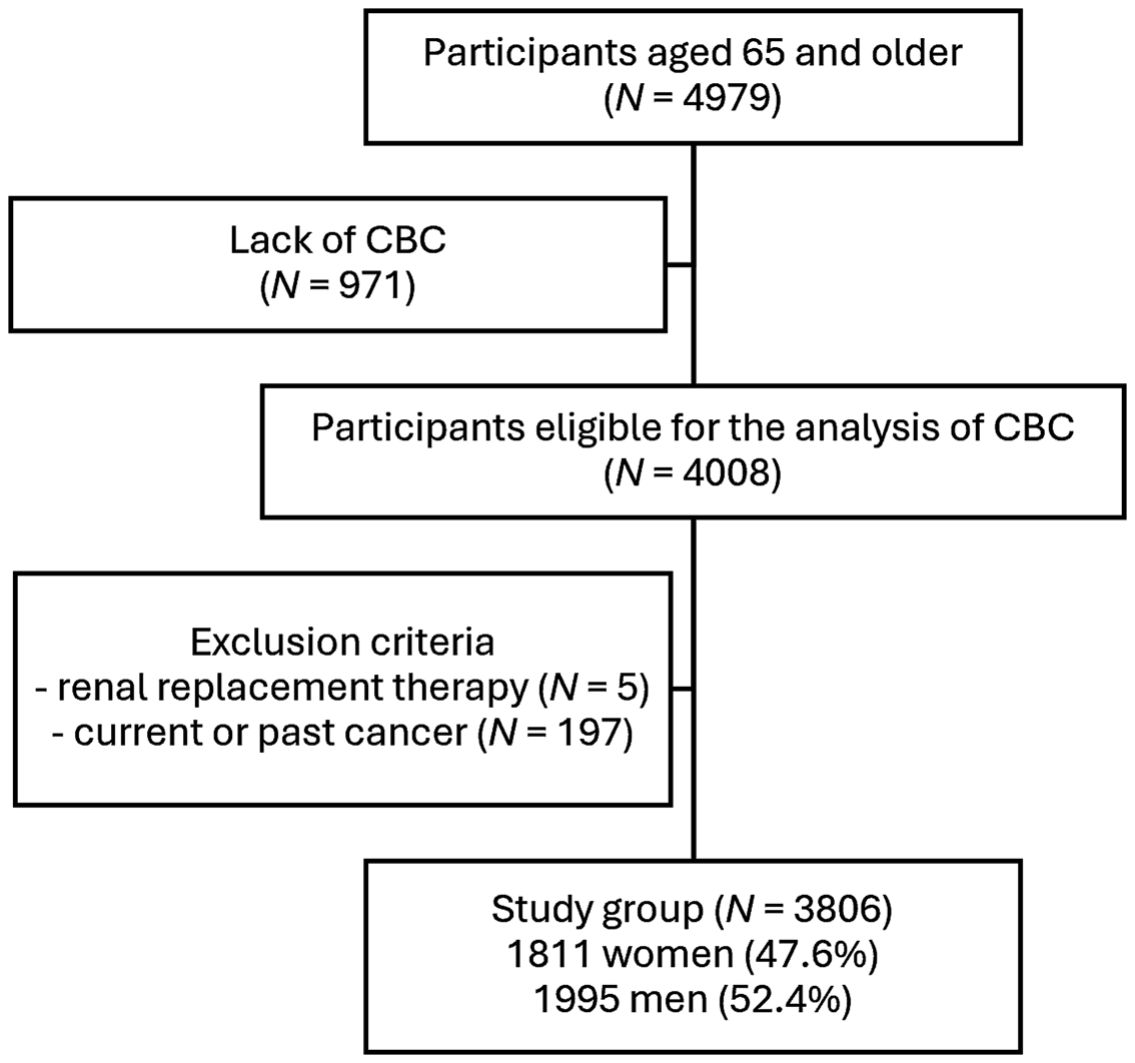
Flow chart. CBC, complete blood count.

Participants’ survival data were obtained from the population register. Access to the database in 2022 revealed that 2,167 (56.9%) of PolSenior participants died, on average, 9.4 ± 0.5 years after joining the study. All evaluations were conducted in accordance with relevant guidelines and regulations, with individuals providing informed consent. The Bioethics Committee of the Medical University of Silesia approved the study protocol (KNW-6501-38/I/08). Standards for biochemical measurements and definitions of comorbidities were previously described (17).

### 2.2 Data analysis

Participants were categorized by sex and divided into six groups based on hemoglobin levels, increasing by 1 g/dL. For females, the groups were as follows: < 11.0 g/dL, 11.0–11.99 g/dL, 12.0–12.99 g/dL, 13.0–13.99 g/dL, 14.0–14.99 g/dL, and ≥ 15.0 g/dL. Males were classified into: < 12.0 g/dL, 12.0–12.99 g/dL, 13.0–13.99 g/dL, 14.0–14.99 g/dL, 15.0–15.99 g/dL, and ≥ 16.0 g/dL. The reference groups for females and males were established at hemoglobin levels of 14.0–14.99 g/dL (*N* = 362) and 15.0–15.99 g/dL (*N* = 374), respectively, as these groups had the lowest mortality rates. The analysis incorporated sociodemographic data, including sex, age, and factors from the comprehensive geriatric assessment. This assessment encompassed activities of daily living (ADL, score 0–6, with values ≤ 4 indicating dependence), instrumental activities of daily living (IADL, score 8–24, with values ≤ 18 indicating dependence), fall incidence in the past year, ability to walk 200 meters, mini-nutritional assessment – short form (MNA-SF, score 0–14), comorbidities such as obesity, diabetes (DM), hypertension, chronic kidney disease, history of stroke, coronary artery disease, hospitalization due to heart failure (HF), atrial fibrillation (AF), peptic ulcer, asthma or chronic obstructive pulmonary disease (COPD), mild cognitive impairment (MCI), dementia, and depression. Additionally, laboratory results included levels of interleukin-6 (IL-6) and C-reactive protein (CRP), and medications such as statins, aspirin, angiotensin-converting enzyme inhibitors (ACE-I), angiotensin receptor blockers (ARB), beta-blockers, thiazide-like diuretics, loop diuretics, spironolactone, and antidepressants. Lastly, smoking history (both past and current) and frequency of alcohol consumption in the past year were also recorded.

### 2.3 Metabolic diseases – definitions

- Obesity – body mass index (BMI) ≥ 30 kg/m^2^ (18)
- Diabetes – serum glucose levels on fasting > 125 mg/dL (19)
- Hypertension – mean values of the two consecutive measurements of ≥ 140 mmHg for systolic and ≥ 90 mmHg for diastolic blood pressure (20)
- Chronic Kidney Disease – estimated glomerular filtration rate (eGFR) using the full Modification of Diet in Renal Disease (MDRD) formula < 45 mL/min/1.73 m^2^; although current criteria for the diagnosis of CKD in adults include persistent signs of kidney damage, or a GFR below 60 mL/min/1.73 m^2^, the latest reports indicate that, unlike in young adults where mortality associated with impaired renal function increases already at GFR < 75 mL/min/1.73 m^2^, in older adults it increases only at GFR < 45 mL/min/1.73 m^2^ (21)

### 2.4 Statistical analysis

Statistical analyses were performed using STATISTICA 13.0 PL (TIBCO Software Inc., Palo Alto, CA, USA), StataSE 13.0 (StataCorp LP, TX, USA), and the R software R version 4.4.0 (R Core Team (2013), R Foundation for Statistical Computing, Vienna, Austria, http://www.R-project.org/). Statistical significance was defined as a *p*-value < 0.05. All tests were two-tailed. Imputations were not performed for missing data. Nominal and ordinal data were expressed as percentages. Interval data were expressed as the mean value ± standard deviation in the case of normal distribution. For data with a skewed or non-normal distribution, they were expressed as the median with lower and upper quartiles. The distribution of variables was evaluated by the Anderson–Darling test and the quantile-quantile (Q-Q) plot. The homogeneity of variances was assessed by the Levene test. Nominal and ordinal data were compared with the χ^2^ test. Comparisons between groups for interval data were conducted using a one-way analysis of variance with Dunnett’s *post hoc* test.

The risk of death according to the hemoglobin level was evaluated with the multivariable stepwise best Cox proportional hazard regression stratified to age and glomerular filtration rate and shown with hazard ratios (HR) and corresponding confidence intervals (±95% CI) and *p*-values. Overall mortality risk factors were assessed with a Pointwise Nonparametric Estimation of Hazard Ratio method (R package ‘*smoothHR*’). This method provides flexible hazard ratio curves that allow non-linear relationships between continuous predictors and survival, as well as a better understanding of the effects of each continuous covariate on the outcome. Results are expressed as hazard ratio curves, with a specific covariate value as the reference. Results are presented with hazard ratios and graphically with smooth HR plots. The proportionality assumption was tested based on the Schoenfeld residuals (R function ‘*cox.zph*’). Multiple-collinearity was checked based on the correlation matrix of coefficients of the survival model. Additionally, overall survival analyses were performed with Kaplan–Meyer curves, stratified by hemoglobin levels and sex, with the log-rank test to compare survival curves.

The hemoglobin cut-off levels as predictors of increased mortality were determined using receiver operating characteristic (ROC) curve analysis, with Youden’s index calculated as sensitivity + specificity – 1. The accuracy of the test was assessed based on the area under the ROC curve (AUC). The AUC values > 0.9, 0.7 to 0.9, and 0.5 to 0.7 corresponded to the high, moderate, and low diagnostic accuracy of the test, respectively (22).

## 3 Results

### 3.1 Baseline study group characteristics

Study groups’ characteristics are presented in Table 1 (women) and Table 2 (men). The median age was 79 (72–86) years; 78 (71–86) for women and 79 (72–86) for men. Mean hemoglobin was 13.2 ± 1.3 g/dL for women and 14.0 ± 1.5 g/dL for men. Subgroups with the lowest hemoglobin levels were older, had higher rates of malnutrition, heart failure, CKD (eGFR < 45 mL/min/1.73 m^2^), past stroke, dementia, dependency on ADL and IADL, falls, and inability to walk 200 m, as well as greater use of loop diuretics and spironolactone in both sexes. Conversely, obesity (BMI ≥ 30 kg/m^2^) was less prevalent in these subgroups. In women, depression was more common with lower hemoglobin. Men with lower hemoglobin exhibited lower rates of hypertension, subjective health status, alcohol consumption, and higher CRP and IL-6 levels. Additionally, women with the highest hemoglobin levels (Hb ≥ 15 g/dL) had greater occurrences of past stroke, dementia, IADL dependence, and spironolactone use.

**Table 1:** The characteristics of the female hemoglobin level subgroups (*N* = 1811; 47.6%).

Hemoglobin level (g/dL)	<11	<11;12)	<12;13)	<13;14)	<14;15)	≥15	*p*
*N* (%)	80 (4.4)	166 (9.2)	432 (23.9)	629 (34.7)	362 (20.0)	142 (7.8)	—
Age (years) (lower – upper quartile)	88 *** **(81–91)**	84 *** **(75–90)**	80 *** **(72–87)**	76 * **(70–85)**	74 (70–81)	78 * **(72–85)**	**<0.001**
Smoking, past or current, *N* (%)	10 (12.5)	25 (15.1)	69 (16.0)	104 (16.5)	76 (21.0)	41 (28.9)	**<0.01**
Alcohol, *N* (%)	6 (7.5)	5 (3.0) *	22 (5.1) *	29 (4.6) **	32 (8.8)	6 (4.2)	**<0.05**
Diabetes, *N* (%)	24 (30.0)	51 (30.7)	102 (23.6)	148 (23.5)	86 (23.8)	33 (23.2)	0.36
Hypertension, *N* (%)	60 (75.0)	128 (77.1)	327 (75.7)	499 (79.3)	287 (79.3)	113 (79.6)	0.69
Past stroke, *N* (%)	11 (13.8) **	21 (12.7) **	23 (5.3)	41 (6.5)	18 (5.0)	19 (13.4) **	**<0.001**
Coronary artery disease, *N* (%)	16 (20.0)	35 (21.1)	97 (22.5)	114 (18.1)	67 (18.5)	22 (15.5)	0.40
Past myocardial infarction, *N* (%)	9 (11.3)	27 (16.3)	79 (18.3)	88 (14.0)	50 (13.8)	16 (11.8)	0.20
Heart failure, *N* (%)	16 (20.0) ***	13 (7.8) **	30 (6.9) **	18 (2.9)	9 (2.5)	7 (4.9)	**<0.001**
Atrial fibrillation, *N* (%)	10 (12.5)	37 (22.3)	90 (20.8)	104 (16.5)	64 (17.7)	29 (20.4)	0.21
Asthma/COPD, *N* (%)	16 (20.0)	22 (13.3)	71 (16.4)	94 (14.9)	61 (16.9)	29 (20.4)	0.46
Ulcer disease, *N* (%)	6 (7.5)	11 (6.6)	37 (8.6)	51 (8.1)	36 (9.9)	10 (7.0)	0.80
MCI, *N* (%)	23 (28.8)	57 (34.3)	146 (33.8)	243 (38.6)	137 (37.8)	40 (28.2)	0.10
Dementia, *N* (%)	36 (45.0) ***	74 (44.6) ***	147 (34.0) **	182 (28.9)	86 (23.8)	52 (36.6) **	**<0.001**
Depression, *N* (%)	31 (38.8) *	65 (39.2) **	159 (36.8) **	197 (31.3)	96 (26.5)	50 (35.2)	**<0.05**
Subjective health status <6, *N* (%)	40 (50.0)	78 (47.0)	212 (49.1)	285 (45.3)	145 (40.1)	66 (46.5)	0.19
Falls, *N* (%)	36 (45.0) ***	58 (34.9) **	115 (26.6)	140 (22.3)	83 (22.9)	32 (22.5)	**<0.001**
<200 m distance walk, *N* (%)	45 (56.3) ***	77 (46.4) ***	168 (38.9) **	208 (33.1)	107 (29.6)	53 (37.3)	**<0.001**
ADL ≤ 4 pts., *N* (%)	28 (35.0) ***	31 (18.7) **	66 (15.3) *	64 (10.2)	36 (9.9)	17 (12.0)	**<0.001**
IADL ≤ 18 pts., *N* (%)	50 (62.5) ***	68 (41.0) ***	140 (32.4) ***	163 (25.9) **	65 (18.0)	41 (28.9) **	**<0.001**
BMI (kg/m^2^) mean ± SD	27.9 ± 6.0 *	27.4 ± 5.4 ***	28.3 ± 5.5 ***	29.4 ± 5.5	29.7 ± 5.3	29.8 ± 5.3	**<0.001**
Obesity (BMI ≥ 30 kg/m^2^), *N* (%)	20 (30.3) ***	45 (29.4) ***	131 (33.4) ***	240 (40.3) *	162 (46.7)	64 (47.4)	**<0.001**
MNA 8–11 pts (at risk), *N* (%)	32 (40.0)	93 (56.0) **	173 (40.0)	260 (41.3)	151 (41.7)	50 (35.2)	**<0.01**
MNA ≤ 7 pts. (malnourished), *N* (%)	20 (25.0) ***	**23 (13.9)**	77 (17.8) ***	59 (9.4)	33 (9.1)	17 (12.0)	**<0.001**
eGFR < 45 mL/min/1.73 m^2^, *N* (%)	24 (30.0) ***	41 (24.7) ***	73 (16.9) ***	61 (9.7)	35 (9.7)	16 (11.3)	**<0.001**
eGFR (mL/min/1.73 m^2^) mean ± SD	52.2 ± 25.2 ***	57.5 ± 19.0 ***	61.6 ± 18.7 **	66.5 ± 16.3	66.3 ± 16.2	63.8 ± 16.4	**<0.001**
CRP (mg/dL) (lower – upper quartile)	2.9 (1.3–7.5)	2.5 (1.2–5.5)	2.3 (1.1–5.2)	2.4 (1.3–4.6)	2.4 (1.2–4.2)	2.7 (1.3–5.4)	0.82
IL-6 (pg/mL) (lower – upper quartile)	**3.8** (2.2–7.3) ***	2.4 (1.6–4.6)	2.4 (1.5–4.1)	2.1 (1.4–3.4)	2.1 (1.4–3.3)	2.3 (1.6–3.8)	**<0.001**
Statins, *N* (%)	15 (19.0)	43 (26.4)	98 (23.1)	156 (25.3)	100 (28.4)	29 (20.7)	0.32
Aspirin, *N* (%)	26 (32.9)	59 (36.4)	147 (34.6)	213 (34.5)	92 (25.9)	51 (36.4)	0.06
ACE-I/ARB, *N* (%)	34 (43.0)	92 (56.4)	210 (49.4)	308 (49.9)	174 (49.0)	68 (48.6)	0.48
Beta-blockers, *N* (%)	29 (36.7)	75 (46.0)	174 (40.9)	233 (37.8)	147 (41.4)	58 (41.4)	0.46
Thiazide-like diuretics, *N* (%)	17 (21.5)	44 (27.0)	103 (24.2)	164 (26.6)	101 (28.5)	40 (28.6)	0.68
Loop diuretics, *N* (%)	17 (21.5) ***	25 (15.3) **	50 (11.8)	37 (6.0)	28 (7.9)	14 (10.0)	**<0.001**
Spironolactone, *N* (%)	14 (17.7) **	21 (12.9) *	50 (11.8) *	40 (6.5)	24 (6.8)	20 (14.3) **	**<0.001**
Antidepressants, *N* (%)	7 (8.9)	16 (9.8)	42 (9.9)	67 (10.9)	35 (9.9)	20 (14.3)	0.72
5 years death, *N* (%)	53 (67.1)	70 (42.2)	142 (32.9)	151 (24.0)	82 (22.6)	41 (28.9)	**< 0.001**
10 years death, *N* (%)	52 (79.7)	111 (66.9)	238 (55.2)	270 (42.9)	137 (37.8)	69 (48.6)	**< 0.001**
Lower quartile overall survival (years) ± 95% CI	1.2 (0.7–1.7)	2.6 (1.7–3.1)	3.6 (3.1–4.4)	5.2 (4.3–5.9)	5.4 (4.4–6.5)	4.6 (3.1–5.5)	–
Median overall survival (years) ± 95% CI	2.9 (2.1–4.6)	6.1 (4.9–7.6)	8.3 (7.2–9.3)	–	–	–	–
Restricted mean (years) ± 95% CI	4.2 (3.5–5.0)	6.0 (5.4–6.5)	6.9 (6.5–7.2)	7.7 (7.5–7.9)	7.8 (7.5–8.2)	7.3 (6.7–7.8)	–
Death incidence rate/1,000 ± 95% CI	196.4 (153.4–251.4)	115.1 (95.5–138.6)	83.5 (73.6–94.8)	58.3 (51.7–65.7)	50.5 (42.7–59.7)	69.8 (55.1–88.3)	–
HR ± 95% CI	**4.01** ^***^ **(2.97–5.41)**	**2.31** ^***^ **(1.79–2.96)**	**1.66** ^***^ **(1.35–2.05)**	1.16 (0.94–1.42)	Ref.	1.39^*^ (1.04–1.85)	–

*p < 0.05; ** *p* < 0.01; ***p < 0.001 vs hemoglobin range <14;15). Statistically significant values are presented in bold.

COPD, chronic obstructive pulmonary disease; MCI, mild cognitive impairment; ADL, activities of daily living; IADL, instrumental activities of daily living; BMI, body mass index; MNA, mini nutritional assessment; eGFR, estimated glomerular filtration rate; CRP, C-reactive protein; IL-6, interleukin-6; ACE-I, angiotensin converting enzyme-inhibitors; ARB, angiotensin receptor blockers.

**Table 2:** The characteristics of the male hemoglobin level subgroups (*N* = 1995; 52.4%).

Hemoglobin level (g/dL)	<12	<12;13)	<13;14)	<14;15)	<15;16)	≥16	*p*
*N* (%)	163 (8.2)	243 (12.2)	483 (24.2)	562 (28.2)	374 (18.7)	170 (8.5)	–
Age (years) (lower – upper quartile)	86 *** **(81–91)**	86 *** **(79–90)**	81 *** **(74–87)**	76 (71–84)	74 (70–80)	74 (69–79)	**<0.001**
Smoking, past or current, *N* (%)	104 (63.8)	145 (59.7)	329 (68.1)	374 (66.5)	254 (67.9)	123 (72.9)	0.10
Alcohol, *N* (%)	23 (14.1) ***	37 (15.2) ***	113 (23.4) **	142 (25.3) *	123 (32.9)	60 (35.3)	**<0.001**
Diabetes, *N* (%)	31 (19.0)	52 (21.4)	100 (20.7)	123 (21.9)	76 (20.3)	34 (20.0)	0.97
Hypertension, *N* (%)	88 (54.0) ***	159 (65.4)	334 (69.2)	400 (71.2)	261 (69.8)	130 (76.5)	**<0.001**
Past stroke, *N* (%)	24 (14.7) ***	28 (11.5) **	39 (8.1)	56 (10.0) *	21 (5.6)	13 (7.6)	**<0.01**
Coronary artery disease, *N* (%)	38 (23.3)	73 (30.0)	119 (24.6)	115 (20.5)	90 (24.1)	31 (18.2)	**<0.05**
Past myocardial infarction, *N* (%)	25 (15.3)	43 (17.7)	64 (13.3)	62 (11.0)	54 (14.4)	20 (11.8)	0.16
Heart failure, *N* (%)	23 (14.1) ***	27 (11.1) **	31 (6.4)	20 (3.6)	17 (4.5)	4 (2.4)	**<0.001**
Atrial fibrillation, *N* (%)	26 (16.0)	44 (18.1)	82 (17.0)	71 (12.6)	54 (14.4)	29 (17.1)	0.28
Asthma/COPD, *N* (%)	41 (25.2)	43 (17.9)	115 (24.1)	91 (16.3)	77 (20.7)	22 (13.1) *	**<0.01**
Ulcer disease, *N* (%)	21 (13.1)	25 (10.4)	52 (10.9)	62 (11.1)	37 (10.1)	13 (7.8)	0.74
MCI, *N* (%)	51 (31.3)	94 (38.7)	173 (35.8)	205 (36.5)	143 (38.2)	70 (41.2)	0.49
Dementia, *N* (%)	62 (38.0) ***	82 (33.7) ***	143 (29.6) **	135 (24.0)	78 (20.9)	39 (22.9)	**<0.001**
Depression, *N* (%)	47 (28.8)	59 (24.3)	122 (25.3)	136 (24.2)	70 (18.7)	40 (23.5)	0.14
Subjective health status <6, *N* (%)	75 (46.0) ***	111 (45.7) ***	211 (43.7) *	208 (37.0)	134 (35.8)	45 (26.5)	**<0.001**
Falls, *N* (%)	49 (30.1) ***	63 (25.9) ***	104 (21.5) ***	93 (16.5)	46 (12.3)	15 (8.8)	**<0.001**
<200 m distance walk, *N* (%)	71 (38.5) ***	92 (37.9) ***	166 (34.4) **	147 (26.2)	92 (24.6)	31 (18.2)	**<0.001**
ADL ≤ 4 pts., *N* (%)	41 (25.2) ***	42 (17.3) ***	59 (12.2) **	37 (6.6)	21 (5.6)	6 (3.5)	**<0.001**
IADL ≤ 18 pts., *N* (%)	85 (52.1) ***	106 (43.6) ***	125 (25.9) *	122 (21.7)	75 (20.1)	27 (15.9)	**<0.001**
BMI (kg/m^2^) mean ± SD	25.7 ± 4.4 ***	26.6 ± 4.3 ***	27.0 ± 4.4 ***	27.6 ± 4.3	28.1 ± 4.5	29.0 ± 4.5 *	**<0.001**
Obesity (BMI ≥ 30 kg/m^2^), *N* (%)	17 (12.3) ***	44 (19.4) ***	107 (23.4) **	154 (28.1)	114 (31.1)	58 (34.5)	**<0.001**
MNA 8–11 pts (at risk), *N* (%)	71 (43.6) ***	90 (37.0) *	182 (37.7) **	205 (36.5) **	105 (28.1)	49 (28.8)	**<0.01**
MNA ≤ 7 pts. (malnourished), *N* (%)	25 (15.3) ***	29 (11.9) **	36 (7.5)	24 (4.3)	22 (5.9)	6 (3.5)	**<0.001**
eGFR < 45 mL/min/1.73 m^2^, *N* (%)	59 (36.2) ***	50 (20.6) ***	48 (9.9) **	27 (4.8)	18 (4.8)	11 (6.5)	**<0.001**
eGFR (mL/min/1.73 m^2^) mean ± SD	54.5 ± 21.2 ***	60.5 ± 18.8 ***	66.7 ± 17.6 ***	71.5 ± 16.9	72.1 ± 17.8	70.4 ± 18.3	**<0.001**
CRP (mg/dL) (lower – upper quartile)	3.6 *** **(1.7–9.7)**	2.7 ** **(1.1–7.0)**	**2.4** **(1.0–5.2)**	2.0 (1.0–4.3)	2.1 (1.0–4.2)	1.6 (0.9–3.9)	**<0.001**
IL-6 (pg/mL) (lower – upper quartile)	3.7 *** **(2.4–8.1)**	3.0 *** **(1.9–4.6)**	2.6 ** **(1.7–4.4)**	2.2 (1.5–3.4)	2.1 (1.3–3.5)	2.3 (1.4–3.5)	**<0.001**
Statins, *N* (%)	37 (23.3)	55 (22.6)	122 (25.6)	129 (23.3)	81 (22.0)	36 (21.3)	0.82
Aspirin, *N* (%)	52 (32.7)	81 (33.6)	175 (36.8)	199 (36.0)	119 (32.3)	55 (32.5)	0.71
ACE-I/ARB, *N* (%)	70 (44.0)	107 (44.4)	228 (47.9) *	223 (40.3)	144 (39.1)	63 (37.3)	**<0.05**
Beta-blockers, *N* (%)	56 (35.2)	84 (34.9)	139 (29.2)	168 (30.4)	108 (29.3)	52 (30.8)	0.51
Thiazide-like diuretics, *N* (%)	18 (11.3)	37 (15.4)	92 (19.3)	95 (17.2)	61 (16.6)	38 (22.5)	0.10
Loop diuretics, *N* (%)	30 (18.9) ***	42 (17.4) ***	49 (10.3)	31 (5.6)	26 (7.1)	5 (3.0)	**<0.001**
Spironolactone, *N* (%)	25 (15.7) **	25 (10.4)	48 (10.1)	30 (5.4)	27 (7.3)	11 (6.5)	**<0.001**
Antidepressants, *N* (%)	8 (5.0)	11 (4.6)	19 (4.0)	25 (4.5)	18 (4.9)	6 (3.6)	0.97
5 years death, *N* (%)	110 (67.5)	147 (60.5)	197 (41.0)	173 (30.8)	98 (26.2)	43 (25.3)	**<0.001**
10 years death, *N* (%)	148 (90.8)	207 (85.2)	324 (67.4)	311 (55.3)	188 (50.3)	89 (52.3)	**<0.001**
Lower quartile overall survival (years) ± 95% CI	1.3 (1.0–1.7)	1.9 (1.4–2.2)	2.8 (2.3–3.4)	4.3 (3.8–4.6)	4.7 (3.8–5.6)	4.6 (3.9–6.3)	–
Median overall survival (years) ± 95% CI	3.2 (2.7–4.0)	3.7 (3.0–4.4)	6.6 (6.0–7.1)	8.2 (7.2–9.2)	9.6 (8.3 – NA)	8.8 (7.5 – NA)	–
Restricted mean (years) ± 95% CI	4.0 (3.5–4.4)	4.6 (4.2–5.0)	6.1 (5.8–6.5)	7.0 (6.8–7.3)	7.3 (7.0–7.7)	7.4 (7.0–7.9)	–
Death incidence rate/1,000 ± 95% CI	231.9 (197.4–272.4)	189.0 (164.9–216.5)	113.1 (101.4–126.1)	81.6 (73.0–91.2)	71.5 (61.9–82.4)	73.2 (59.5–90.1)	–
HR ± 95% CI	**3.52** ^***^ **(2.83–4.37)**	**2.81** ^***^ **(2.31–3.43)**	**1.62** ^***^ **(1.35–1.92)**	1.15 (0.96–1.37)	Ref.	1.02 (0.80–1.32)	–

*p < 0.05; **p < 0.01; ***p < 0.001 vs hemoglobin range <15;16). Statistically significant values are presented in bold.

COPD, chronic obstructive pulmonary disease; MCI, mild cognitive impairment; ADL, activities of daily living; IADL, instrumental activities of daily living; BMI, body mass index; MNA, mini nutritional assessment; eGFR, estimated glomerular filtration rate; CRP, C-reactive protein; IL-6, interleukin-6; ACE-I, angiotensin converting enzyme-inhibitors; ARB, angiotensin receptor blockers. NA - not applicable.

### 3.2 Outcome data

During a 9.4 ± 0.5 years follow-up, 2,167 (56.9%) PolSenior participants died. Death rates were higher in men at 63.7% (95% CI: 61.6–65.9%) than in women at 49.4% (95% CI: 47.2–51.7%); *p* < 0.001. Death frequency increased linearly by age: from 15.4% in those aged 65–69 to 96.3% in women ≥90 years, and from 29.9 to 97.4% in men, respectively. Mortality increased by 16.2% in women and 13.5% in men per age stratum.

The mean hemoglobin level in women who died was 13.0 ± 1.5 g/dL, lower than in survivors (13.4 ± 1.1 g/dL; *p* < 0.001). In men, the mean hemoglobin level among those who died was 13.7 ± 1.6 g/dL, which was lower than that of survivors (14.6 ± 1.2 g/dL; *p* < 0.001). Kaplan–Meier analysis in women showed that those with levels < 13 g/dL had higher mortality over time than those with 14–15 g/dL (*p* < 0.01) – Figure 2A. The cohort with levels ≥ 15 g/dL also had increased mortality compared to the 14–15 g/dL group (*p* < 0.05). No difference in mortality was observed between cohorts with levels of 13–14 g/dL and 14–15 g/dL (*p* = 0.19). For men, Kaplan–Meier analysis indicated that those with levels < 14 g/dL had increased mortality over time compared to those with levels 15–16 g/dL (*p* < 0.01) – Figure 2B. No mortality difference was noted between those ≥ 14 g/dL. A significant (*p* < 0.001) trend in 5-year and 10-year mortality was observed from the highest to the lowest hemoglobin levels for all genders.

**Figure 2:**
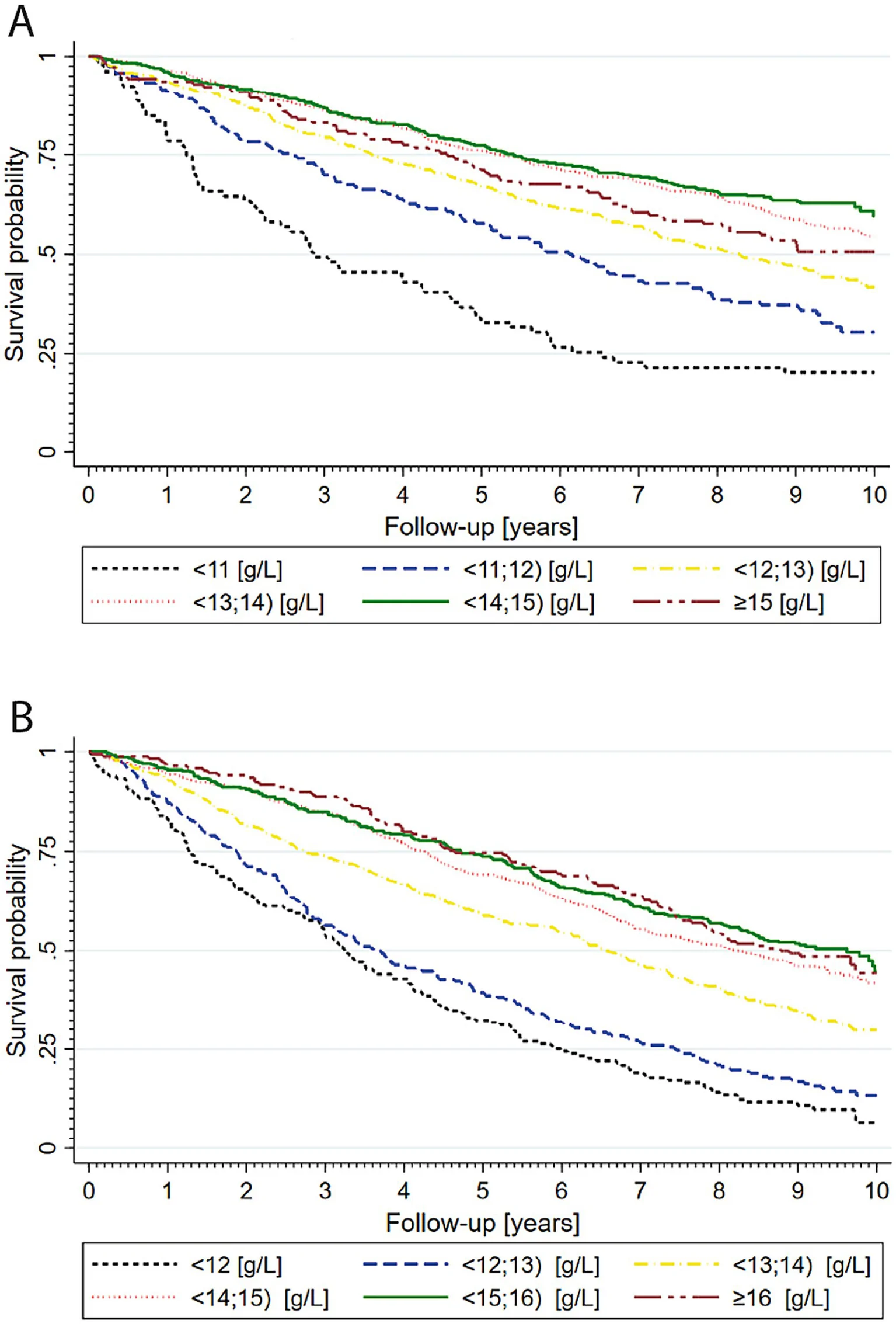
Kaplan–Meier survival estimates according to hemoglobin level cohorts **(A)** Survival estimates for women; compared with the cohort with hemoglobin levels between 14 and 15 g/dL, the cohorts with hemoglobin levels lower than 13 g/dL, as well as the cohort with hemoglobin levels equal to or higher than 15 g/dL, had increased all-cause mortality over time (*p* < 0.01 and *p* < 0.05, respectively) **(B)** Survival estimates for men; compared with the cohort with hemoglobin levels between 15 and 16 g/dL, the cohorts with hemoglobin levels lower than 14 g/dL, had increased all-cause mortality over time (*p* < 0.01).

### 3.3 Estimation of hemoglobin level cut-off points for increased mortality

The receiver operating characteristic (ROC) analysis indicated that a hemoglobin level below 13.3 g/dL weakly predicted increased mortality in women, with an accuracy of 58.1% and AUC = 0.604 (95% CI: 0.578–0.630), a positive predictive value (PPV) of 57.8% (95% CI: 54.5–61.1%), and a negative predictive value (NPV) of 58.3% (95% CI: 55.1–61.5%). Similarly, a hemoglobin level below 13.9 g/dL weakly predicted increased mortality in men, with an accuracy of 60.2% and AUC = 0.665 (95% CI: 0.641–0.689), a PPV of 76.8% (95% CI: 73.9–79.5%), and an NPV of 53.2% (95% CI: 50.2–56.1%).

Table 3 presents characteristics of subgroups divided by hemoglobin cutoffs (13.3 g/dL for women and 13.9 g/dL for men). Women and men with hemoglobin below the cut-off were significantly older than those with levels at or above the cut-off. There was a higher prevalence of malnutrition, heart failure, CKD, dementia, depression, dependence on ADL and IADL, falls, inability to walk 200 m, low subjective health status, and use of loop diuretics and spironolactone in groups with hemoglobin below the cut-off. Conversely, the percentage of obese individuals (BMI ≥ 30 kg/m^2^) was lower in these subgroups. Fewer women smoked in the lower hemoglobin group. Men below cut-offs had a lower prevalence of hypertension, alcohol consumption, and higher CRP and IL-6 levels, as well as a higher prevalence of asthma/COPD, atrial fibrillation, and use of ACE-I/ARB.

**Table 3:** The characteristics of the subgroups stratified by hemoglobin level (13.3 g/dL for the women and 13.9 g/dL for the men).

Hemoglobin level (g/dL)	Women (*N* = 1811; 47.6%)			Men (*N* = 1995; 52.4%)		
	< 13.3	≥ 13.3	*p*	< 13.9	≥ 13.9	*p*
*N* (%)	874 (48.3)	937 (51.7)	–	889 (44.6)	1,106 (55.4)	
Hemoglobin (g/dL) (lower – upper quartile)	12.4 (11.8–12.9)	14.0 (13.6–14.6)	–	13.1 (12.3–13.5)	14.9 (14.4–15.6)	–
Age (years) (lower – upper quartile)	81 (72–88)	76 (70–84)	**<0.001**	84 (76–89)	75 (70–82)	**<0.001**
Smoking, past or current, *N* (%)	129 (14.9)	96 (21.0)	**<0.001**	578 (65.2)	751 (68.2)	0.16
Alcohol, *N* (%)	45 (5.2)	55 (6.0)	0.49	173 (19.7)	325 (29.8)	**<0.001**
Diabetes, *N* (%)	224 (26.1)	220 (23.7)	0.23	183 (20.9)	233 (21.5)	0.76
Hypertension, *N* (%)	674 (77.7)	740 (79.5)	0.34	581 (65.7)	791 (71.8)	**<0.01**
Past stroke, *N* (%)	63 (7.2)	70 (7.5)	0.83	91 (10.3)	90 (8.2)	0.11
Coronary artery disease, *N* (%)	181 (20.7)	170 (18.1)	0.17	230 (25.9)	236 (21.3)	**<0.05**
Past myocardial infarction, *N* (%)	138 (15.8)	131 (14.0)	0.28	132 (15.4)	136 (12.7)	0.09
Heart failure, *N* (%)	68 (8.1)	25 (2.8)	**<0.001**	81 (9.4)	41 (3.8)	**<0.001**
Atrial fibrillation, *N* (%)	169 (19.3)	165 (17.6)	0.34	152 (18.5)	154 (14.7)	**<0.05**
Asthma/COPD, *N* (%)	142 (16.2)	151 (16.1)	0.94	199 (22.6)	190 (17.3)	**<0.01**
Ulcer disease, *N* (%)	65 (7.4)	86 (9.2)	0.18	98 (11.2)	112 (10.3)	0.51
MCI, *N* (%)	302 (36.1)	344 (37.2)	0.63	318 (37.6)	418 (38.6)	0.67
Dementia, *N* (%)	322 (38.5)	255 (27.6)	**<0.001**	287 (34.0)	252 (23.3)	**<0.001**
Depression, *N* (%)	317 (42.3)	381 (33.2)	**<0.001**	228 (29.8)	246 (23.5)	**<0.01**
Subjective health status <6, *N* (%)	419 (53.0)	407 (46.2)	**<0.01**	397 (49.6)	387 (36.1)	**<0.001**
Falls, *N* (%)	257 (29.4)	207 (22.1)	**<0.001**	216 (24.7)	154 (14.0)	**<0.001**
<200 m distance walk, *N* (%)	362 (46.4)	296 (33.6)	**<0.001**	329 (39.9)	270 (25.4)	**<0.001**
ADL ≤ 4 pts, *N* (%)	145 (16.6)	97 (10.4)	**<0.001**	142 (16.0)	64 (5.8)	**<0.001**
IADL ≤ 18 pts., *N* (%)	312 (35.9)	215 (23.0)	**<0.001**	316 (35.8)	224 (20.4)	**<0.001**
BMI (kg/m^2^)	28.3 ± 5.5	29.6 ± 5.4	**<0.001**	26.7 ± 4.4	28.0 ± 4.4	**<0.001**
Obesity (BMI ≥ 30 kg/m^2^), *N* (%)	267 (33.6)	395 (44.2)	**<0.001**	168 (20.4)	326 (30.1)	**<0.001**
MNA 8–11 pts (at risk), *N* (%)	373 (42.7)	386 (41.2)	0.52	343 (43.4)	359 (33.8)	**<0.001**
MNA ≤ 7 pts. (malnourished), *N* (%)	145 (16.6)	84 (9.0)	**<0.001**	90 (11.4)	52 (4.9)	**<0.001**
eGFR (mL/min/1.73 m^2^) mean ± SD	61.1 ± 19.5	66.1 ± 16.3	**<0.001**	62.8 ± 19.2	71.5 ± 17.5	**<0.001**
eGFR < 45 mL/min/1.73 m^2^, *N* (%)	160 (18.9)	90 (9.7)	**<0.001**	157 (18.0)	56 (5.2)	**<0.001**
CRP (mg/dL) (lower – upper quartile)	2.4 (1.1–5.4)	2.5 (1.3–4.5)	0.79	2.7 (1.1–6.3)	2.0 (1.0–4.2)	**<0.001**
IL-6 (pg/mL) (lower – upper quartile)	2.4 (1.6–4.2)	2.2 (1.4–3.4)	**<0.01**	2.9 (1.8–5.1)	2.2 (1.4–3.5)	**<0.001**
Statins, *N* (%)	202 (23.1)	239 (25.5)	0.23	214 (24.1)	246 (22.2)	0.33
Aspirin, *N* (%)	299 (34.6)	289 (31.6)	0.16	308 (35.2)	373 (34.2)	0.66
ACE-I/ARB, *N* (%)	434 (49.7)	452 (48.2)	0.55	405 (45.6)	430 (38.9)	**<0.01**
Beta-blockers, *N* (%)	357 (41.4)	359 (39.2)	0.35	279 (31.8)	328 (30.1)	0.40
Thiazide-like diuretics, *N* (%)	215 (24.9)	254 (27.7)	0.18	147 (16.8)	194 (17.8)	0.55
Loop diuretics, *N* (%)	105 (12.2)	66 (7.2)	**<0.001**	121 (13.8)	62 (5.7)	**<0.001**
Spironolactone, *N* (%)	96 (11.1)	73 (8.0)	**<0.05**	98 (11.2)	68 (6.2)	**<0.001**
Antidepressants, *N* (%)	84 (9.6)	103 (11.0)	0.33	38 (4.3)	49 (4.5)	0.86
5 years death rate, *N* (%)	322 (36.9)	217 (23.1)	**<0.001**	454 (51.2)	314 (28.4)	**<0.001**
10 years death rate, *N* (%)	501 (57.4)	387 (41.3)	**<0.001**	679 (76.6)	588 (53.2)	**<0.001**
Lower quartile overall survival (years) ± 95% CI	3.0 (2.7–3.4)	5.3 (4.8–6.0)	–	2.1 (1.9–2.4)	4.5 (4.1–4.7)	–
Median overall survival (years) ± 95% CI	–	–	–	4.8 (4.4–5.3)	8.6 (8.1–9.6)	–
Restricted mean (years) ± 95% CI	6.6 (6.2–6.8)	7.8 (7.6–8.0)	–	5.3 (5.1–5.5)	7.2 (7.0–7.4)	–
Death incidence rate/1,000 ± 95% CI	90.9 (83.3–99.3)	55.6 (50.3–61.1)	**<0.001**	147.6 (136.9–159.2)	76.8 (70.8–83.3)	**<0.001**
Incidence rate difference/1,000 ± 95% CI	35.3 (25.6–45.0)	–	**<0.001**	70.8 (58.1–83.5)	–	**<0.001**
Incidence rate ratio/HR ± 95% CI	1.63 (1.43–1.87)	–	**<0.001**	1.92 (1.72–2.15)	–	**<0.001**
HR ± 95% CI	1.64 (1.44–1.88)	–	**<0.001**	1.98 (1.78–2.22)	–	**<0.001**

COPD, chronic obstructive pulmonary disease; MCI, mild cognitive impairment; ADL, activities of daily living; IADL, instrumental activities of daily living; BMI, body mass index; MNA, mini nutritional assessment; eGFR, estimated glomerular filtration rate; CRP, C-reactive protein; IL-6, interleukin-6; ACE-I, angiotensin converting enzyme-inhibitors; ARB, angiotensin receptor blockers. Statistically significant values are presented in bold.

Kaplan–Meier analysis revealed that subgroups with hemoglobin levels below the cut-off had higher overall 5- and 10-year mortality rates than those at or above the cut-off (*p* < 0.001).

### 3.4 Non-parametric estimation of hazard ratio curves

The univariable non-parametric survival regression model used hemoglobin as a continuous predictor. Results are shown in Figure 3. The hazard ratio of death within 10 years decreased linearly to 13.3 g/dL for women in both unadjusted and adjusted analyses stratified to age ≥ 80 years and eGFR < 45 mL/min/1.73 m2; this risk is statistically significant compared to values above 13.3 g/dL. For men, the hazard ratio decreased linearly even above the cut-off of 13.9 g/dL in unadjusted analysis. However, adjusted analysis indicated significant risks for hemoglobin levels below 13.9 g/dL for age ≥ 80 years and eGFR < 45 mL/min/1.73 m^2^.

**Figure 3:**
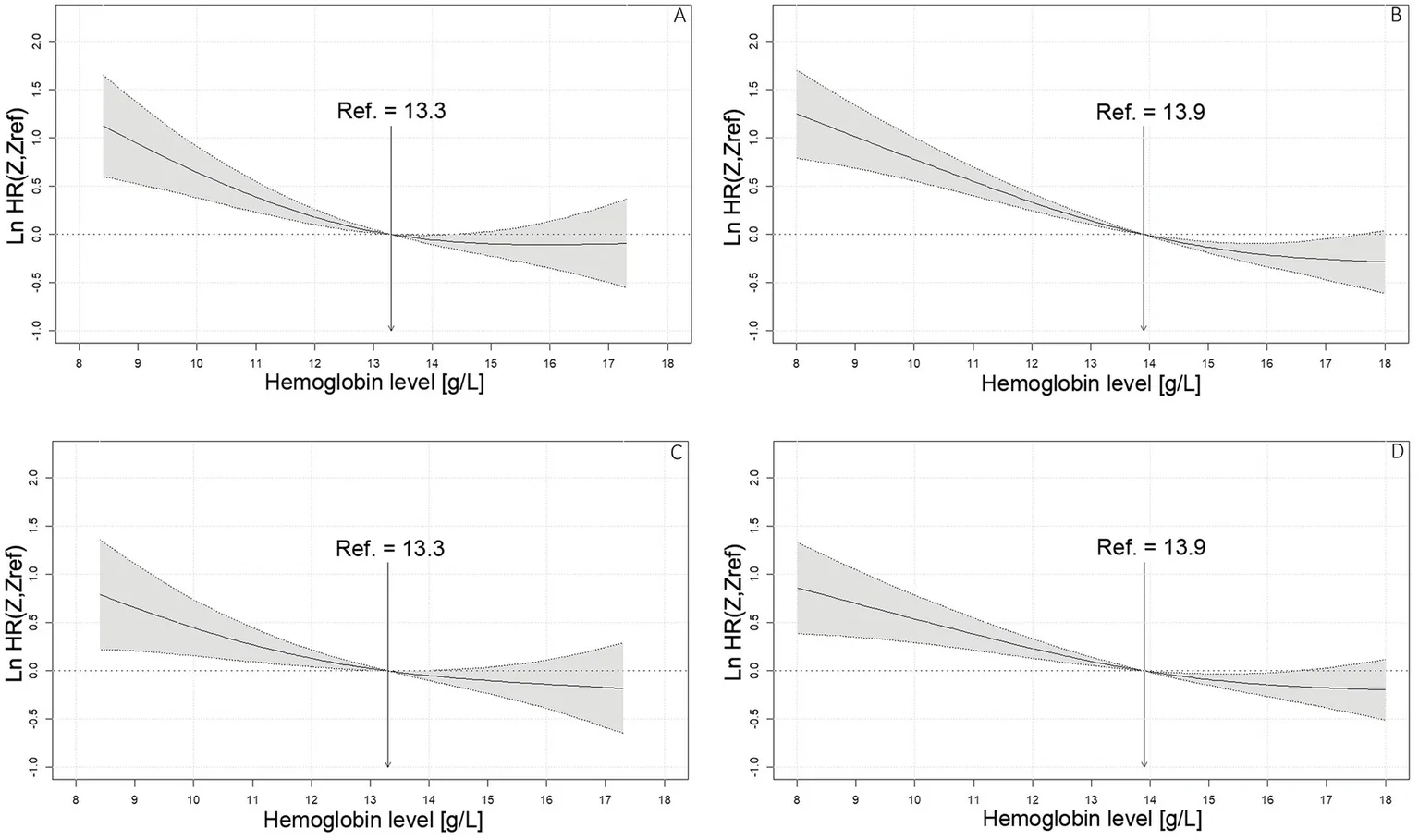
Adjusted and unadjusted nonparametric estimates of the dependence of 10-year risk of death on hemoglobin level stratified to age ≥ 80 and eGFR <45 mL/min/1.73 m^2^ among women and men (log hazard ratio, with 95% confidence limits). **(A)** The crude analysis for women; **(B)** The crude analysis for men **(C)** The adjusted analysis for women **(D)** The adjusted analysis for men. Adjustments were made to age ≥ 80 years and eGFR <45 mL/min/1.73 m^2^.

In the stepwise survival regression model, factors included were hemoglobin level, smoking, obesity (BMI ≥ 30 kg/m^2^), diabetes, hypertension, past stroke, heart failure, atrial fibrillation, asthma/COPD, mild cognitive impairment, dementia, depression, subjective health status below 6, falls, inability to walk 200 m, dependence for instrumental activities of daily living (IADL ≤ 18 + ADL > 4), basic activities of daily living (ADL ≤ 4), malnutrition (MNA ≤ 7), CRP ≥ 3 mg/dL, and IL-6 ≥ 2.4 mg/dL. IL-6 was not included in the multivariable model, as it was set at the median for all genders, while CRP used a cut-off of 3 mg/dL. Low hemoglobin, heart failure, mild cognitive impairment, dementia, inability to walk 200 m, and dependence on ADL/IADL increased the 5-year and 10-year mortality risk for both sexes. The 5-year death risk increased with diabetes for men and with falls for women. The 10-year risk increased with obesity, diabetes, and CRP ≥ 3 mg/dL for men, and with falls for women. Hypertension correlated with a lower 5-year death risk but not a 10-year risk in women. The results are presented in Tables 4, 5.

**Table 4:** Factors affecting the overall survival (univariable Cox proportional hazard), stratified to age ≥80 and eGFR < 45 mL/min/1.73 m^2^.

Variable	10 years				5 years			
	Women		Men		Women		Men	
	HR	± 95% CI	HR	± 95% CI	HR	± 95% CI	HR	± 95% CI
Hemoglobin (g/dL)	**0.90** ^***^	0.86–0.95	**0.86** ^***^	0.83–0.89	**0.87** ^***^	0.82–0.93	**0.84** ^***^	0.80–0.88
Smoking	0.83	0.68–1.01	**1.22** ^**^	1.08–1.38	0.93	0.72–1.21	1.11	0.96–1.30
Diabetes	–	–	**1.22** ^**^	1.06–1.39	**1.25** ^*^	1.03–1.51	**1.33** ^**^	1.12–1.57
Hypertension	–	–	**0.81** ^***^	0.72–0.91	–	–	**0.79** ^**^	0.68–0.91
Past stroke	1.19	0.95–1.50	**1.74** ^***^	1.46–2.07	1.13	0.85–1.51	**1.76** ^***^	1.43–2.17
Heart failure	**2.40** ^***^	1.92–3.02	**2.33** ^***^	1.90–2.85	**2.53** ^***^	1.93–3.12	**2.46** ^***^	1.96–3.08
Atrial fibrillation	1.17	0.99–1.38	**1.16** ^*^	1.01–1.36	1.04	0.84–1.30	1.09	0.89–1.33
Asthma/COPD	1.06	0.89–1.27	**1.19** ^*^	1.04–1.36	1.04	0.83–1.31	1.14	0.96–1.35
MCI (1)	**1.40** ^**^	1.13–1.74	**1.29** ^***^	1.11–1.49	**1.40** ^*^	1.03 – 1.90	**1.31** ^*^	1.06 – 1.61
Dementia (2)	**2.66** ^***^	2.15–3.28	**2.18** ^***^	1.87–2.54	**3.08** ^***^	2.30 – 4.13	**2.60** ^***^	2.12 – 3.19
Depression	**1.98** ^**^	1.11–1.51	**1.36** ^***^	1.20–1.55	**1.31** ^**^	1.07–1.61	**1.46** ^***^	1.24–1.72
Subjective health status <6	1.14	0.99–1.32	**1.24** ^***^	1.10–1.39	1.17	0.97–1.42	**1.26** ^**^	1.08–1.47
Falls	**1.50** ^***^	1.30–1.73	**1.53** ^***^	1.34–1.75	**1.63** ^***^	1.37–1.95	**1.59** ^***^	1.35–1.88
<200 m distance walk	–	–	**1.89** ^***^	1.67–2.13	–	–	**2.05** ^***^	1.76–2.39
IADL ≤ 18 + ADL > 4	**2.64** ^***^	2.22–3.14	**2.15** ^***^	1.86–2.47	**3.29** ^***^	2.63–4.13	**2.41** ^***^	2.02–2.87
IADL ≤ 18 + ADL ≤ 4	**4.11** ^***^	3.41–4.96	**3.83** ^***^	3.23–4.55	**5.38** ^***^	4.24–6.81	**4.23** ^***^	3.48–5.16
Obesity (BMI ≥ 30 kg/m^2^)	**0.81** ^**^	0.69–0.94	0.91	0.79–1.04	0.81*	0.66–0.99	0.85	0.71–1.03
MNA ≤ 7 pts. (malnourished)	**1.45** ^***^	1.22–1.72	**1.72** ^***^	1.43–2.09	**1.50** ^***^	1.21–1.85	**1.75** ^***^	1.39–2.20
CRP ≥ 3 mg/dL	**1.20** ^**^	1.05–1.37	–	–	**1.30** ^**^	1.09–1.54	–	–
IL-6 ≥ 2.4 mg/dL	**1.61** ^***^	1.40–1.86	**1.89** ^***^	1.67–2.14	**1.55** ^***^	1.29–1.87	**2.16** ^***^	1.83–2.56

Results of the univariable Cox regression analysis. *p < 0.05; **p < 0.01; ***p < 0.001. Statistically significant values are presented in bold.

eGFR, estimated glomerular filtration rate; COPD, chronic obstructive pulmonary disease; MCI, mild cognitive impairment, IADL, instrumental activities of daily living; ADL, activities of daily living; BMI, body mass index; MNA, mini nutritional assessment; CRP, C-reactive protein.

**Table 5:** Factors affecting the overall survival (multivariable Cox proportional hazard), stratified to age ≥80 and eGFR <45 mL/min/1.73 m^2^.

Variable	10 years				5 years			
	Women		Men		Women		Men	
	HR	± 95% CI	HR	± 95% CI	HR	± 95% CI	HR	± 95% CI
Hemoglobin (g/dL)	0.92^*^	0.87–0.97	0.89^***^	0.86–0.93	0.90^**^	0.84–0.97	0.89^***^	0.84–0.93
Obesity (BMI ≥ 30 kg/m^2^)	–	–	1.24^**^	1.08–1.41	–	–	–	–
Diabetes	–	–	1.19^*^	1.02–1.38	–	–	1.28^**^	1.07–1.54
Hypertension	–	–	–	–	0.76^*^	0.61–0.96		
Past stroke	–	–	1.34^**^	1.09–1.65	–	–	–	–
Heart failure	1.85^***^	1.41–2.43	2.08^***^	1.67–2.60	1.79^***^	1.31–2.45	2.13^***^	1.67–2.71
MCI	1.34^**^	1.08–1.69	1.17^*^	1.01–1.37	1.33	0.96–1.84	1.21	0.97–1.51
Dementia	1.93^***^	1.53–2.43	1.47^***^	1.24–1.76	1.93^***^	1.40–2.67	1.71^***^	1.36–2.16
Falls	1.24^*^	1.05–1.45	–	–	1.42^**^	1.16–1.74	–	–
<200 m distance walk	1.47^***^	1.23–1.75	1.28^**^	1.11–1.48	1.66^***^	1.30–2.11	1.28^**^	1.07–1.54
IADL ≤ 18 + ADL > 4	1.97^***^	1.62–2.39	1.68^***^	1.41–2.00	2.20^***^	1.71–2.85	1.81^***^	1.47–2.24
IADL ≤ 18 + ADL ≤ 4	2.30^***^	1.79–2.94	2.19^***^	1.73–2.76	2.50^***^	1.83–3.43	2.28^***^	1.75–2.96
CRP ≥ 3 mg/dL	–	–	1.58^***^	1.39–1.78	–	–	1.67^***^	1.42–1.96

Results of the multivariable Cox regression analysis. *p < 0.05; **p < 0.01; ***p < 0.001.

eGFR, estimated glomerular filtration rate; BMI, body mass index; MCI, mild cognitive impairment; IADL, instrumental activities of daily living; ADL, activities of daily living; CRP, C-reactive protein.

## 4 Discussion

The primary objective of this nationwide study was to identify specific hemoglobin thresholds associated with increased 10-year mortality and functional dependency among community-dwelling older adults in Poland. Our prospective analysis revealed a clear, independent relationship between baseline hemoglobin concentrations and long-term overall survival. Crucially, the lowest mortality rates were observed at hemoglobin levels of 14.0–14.9 g/dL for females and 15.0–15.9 g/dL for males. Below these optimal ranges, the risk of death increased progressively. This finding challenges the traditional clinical paradigm that regards mild anemia in the older adults as an “innocent bystander” of the aging process (3). Instead, our data strongly support the growing body of evidence indicating that even a minor, subclinical decline in hemoglobin levels serves as a predictor of adverse health outcomes (5, 6).

Findings from major cohorts such as the Health and Anemia (H&A) study, the Cardiovascular Health Study, and the Established Populations for Epidemiologic Studies of the Elderly (EPESE) have demonstrated that even mild anemia increases the risk of hospitalization and mortality (8–10). However, many earlier studies focused primarily on short- or mid-term survival—such as the three-year follow-up in the H&A cohort or a single year post-hospitalization in regional populations (8). In contrast, our study provides a robust, nationwide assessment spanning a decade, using comprehensive data from the PolSenior project.

While some literature has suggested that low hemoglobin levels, even within the conventional “normal” range, might increase mortality, defining an optimal threshold for community-dwelling seniors remains a subject of ongoing debate. (4, 23). By establishing sex-specific reference groups with the lowest empirical mortality, our study fills this gap by defining the exact inflection points at which survival begins to decline in a large, representative Polish population.

Beyond its significant effect on survival, our study demonstrated an association between low hemoglobin levels and declines in physical and cognitive capacities among older adults. Participants with lower hemoglobin values exhibited significantly higher rates of dependency in both basic activities of daily living (ADL) and instrumental activities of daily living (IADL), alongside an increased incidence of falls and a reduced ability to walk 200 meters. These findings align with earlier observational cohorts, such as those by Penninx et al., which linked late-life anemia to physical disability, diminished muscle strength, and recurrent falls (5, 6).

Importantly, our data also revealed a correlation between lower hemoglobin levels and cognitive impairment (MCI and dementia). This clinical relationship is well-documented globally (24–26). The Bushehr Elderly Health program and the Women’s Health and Aging Study II both highlighted a robust link between mild anemia and executive function deficits in community-dwelling older cohorts (27, 28). Neuroimaging studies suggest that a chronic reduction in oxygen-carrying capacity may exacerbate cerebral white matter hyperintensities, thereby accelerating neurodegenerative and vascular pathways to cognitive decline (29). Another psychogeriatric syndrome proven to be associated with anemia is depression. The ‘Investigations Préventives et Cliniques’ (IPC) cohort study performed in France among adults free of chronic disease and medication from the general population found depressed individuals more likely to have anemia, with prevalence increasing with depression severity, indicating a dose–response relationship (30). The InCHIANTI study in Italy also links anemia and depression (31). Our study supports the association between depression and anemia, as well as a relationship between 5- and 10-year survival and depression in both sexes, confirmed only by univariable logistic regression analysis. By showing that lower hemoglobin levels independently co-occur with both motor and cognitive deterioration, as well as depression, our study underscores hemoglobin’s role as a critical determinant of functional independence in seniors.

Several interrelated pathophysiological pathways can explain the biological mechanisms linking low hemoglobin levels to elevated mortality in older adults. At its core, a decrease in hemoglobin reduces the blood’s oxygen-carrying capacity, leading to chronic tissue hypoxia. In older individuals, whose cardiovascular reserve is already compromised by aging, this hypoxia triggers compensatory increases in cardiac output and heart rate (32). Over time, this chronic hemodynamic stress induces left ventricular hypertrophy, arterial stiffness, and myocardial remodeling, significantly elevating the long-term risk of cardiovascular events and heart failure (33).

Furthermore, during acute illnesses or periods of physiological stress, anemic older adults are highly vulnerable to supply–demand mismatch in vital organs. This can precipitate type 2 myocardial infarction, acute kidney injury, or severe ischemic events (34, 35). On a cellular level, chronic hypoxia and the underlying etiology of anemia — often driven by low-grade chronic inflammation — are intricately intertwined with the development of frailty (36). Pro-inflammatory cytokines, such as interleukin-6 (IL-6) and C-reactive protein (CRP), not only suppress erythropoiesis but also drive sarcopenia and muscle wasting, creating a vicious cycle of anemia, frailty, and accelerated mortality (37).

A long-standing debate in geriatric medicine is whether the higher mortality rates in older adults with low hemoglobin occur independently of underlying pathologies, or if low hemoglobin is merely a surrogate marker for advanced chronic diseases. Previous investigations into unexplained anemia have yielded conflicting results, with some regional studies finding no increased risk of death, while others demonstrated highly cause-specific mortality patterns (12, 13, 38). Our analysis addresses this debate by adjusting for a comprehensive spectrum of geriatric covariates. By accounting for a wide array of confounding variables — including nutritional status (MNA-SF), lifestyle factors, inflammatory markers (IL-6, CRP), and chronic diseases — we confirmed that the survival disadvantage associated with low hemoglobin persists independently of these severe confounding factors. CKD seems most problematic among them, since it significantly increases mortality and concomitantly remains a leading cause of anemia. Thus, we stratified the study population by age and eGFR. In the multivariable logistic regression, factors affecting 10-year survival for both sexes included hemoglobin level, as well as heart failure, mild cognitive impairment, dementia, mobility limitations, and dependence on ADLs and IADLs. Additionally, in women, the factors influencing 10-year overall survival were falls, and in men, obesity, diabetes, previous stroke, and elevated inflammatory markers.

Unexpectedly, our analysis revealed that hypertension improves 5-year survival among women, but no effect was observed on 10-year survival. This finding contrasts with evidence showing that hypertension worsens survival in older adults (39). Notably, multivariable analysis utilized age-stratified data. In our cohort, men and women over 80 experienced lower hypertension prevalence than those aged 65–79. Hypertension prevalence was 80.7% among young-old women, compared with 76.1% among older women (*p* < 0.05). Among men, the difference was larger (73.7% vs. 63.9%; *p* < 0.0001), possibly explaining the lower HR in hypertensive men observed in the univariable analysis. Since advanced age is a stronger mortality predictor than hypertension, the higher prevalence in the younger group may have biased the odds ratio for mortality (40).

From a public health perspective, this study’s findings shift the paradigm of late-life anemia from an individual clinical concern to a significant population-level challenge. As the global population aging accelerates, identifying modifiable risk factors that predict long-term mortality and functional decline is paramount for sustaining public healthcare systems (41, 42). Our sex-specific hemoglobin thresholds (13.3 g/dL for women and 13.9 g/dL for men) that independently predict 10-year mortality provide health policy planners with concrete, empirical metrics for secondary prevention. By demonstrating that even subclinical, low-normal hemoglobin levels co-occur with a cascade of adverse geriatric outcomes—including malnutrition, dementia, and dependency in daily activities—these data suggest that early screening could serve as a cost-effective indicator for broader vulnerability in community-dwelling older adults (8, 42).

Furthermore, these insights have profound implications for designing targeted public health interventions. Traditional public health frameworks often overlook mild anemia, classifying it as a benign surrogate of senescence. However, our multivariable analysis underscores its role as a robust predictor of the loss of physical and cognitive independence (8, 43). Public health strategies should therefore shift from reactive, acute-care treatments to proactive, community-based screening programs embedded in routine geriatric evaluations (41, 44). Implementing systematic hemoglobin monitoring in primary care, alongside nutritional and inflammatory assessments, would allow public health professionals to identify high-risk cohorts before irreversible functional decline and subsequent health system institutionalization occur (8, 44).

Finally, addressing these thresholds aligns with broader socioeconomic strategies to promote healthy aging and reduce the societal burden of multimorbidity. Because low hemoglobin levels independently increase the long-term risk of physical dependency and are associated with a higher incidence of debilitating falls, preventing even mild declines can substantially prolong disability-free life expectancy (42, 43). In countries facing severe demographic shifts, such as Poland, integrating sex-specific hemoglobin surveillance into nationwide senior welfare and public health guidelines could significantly mitigate the escalating economic strain associated with long-term care and repeated hospitalizations for preventable geriatric syndromes (45).

This study has several limitations worth noting. First, the baseline laboratory and clinical data were collected during the initial phase of the PolSenior project (2007–2012), meaning that changes in clinical practice, subsequent therapeutic interventions (such as iron supplementation or blood transfusions), or participants’ fluctuating health statuses during the 10-year follow-up could not be dynamically tracked. Second, while we controlled for an extensive list of comorbidities, the observational nature of the data precludes drawing direct, definitive causal conclusions.

Despite these limitations, the study possesses major strengths, including its nationwide scale, the large and well-characterized cohort of 3,806 participants, and a highly reliable 10-year prospective follow-up linked directly to national population registers, which minimized the risk of missing survival data.

## 5 Conclusions and public health implications

In conclusion, lower hemoglobin levels are a potent, independent predictor of 10-year mortality, cognitive decline, and physical dependency among Polish seniors. These findings strongly discourage lowering reference values for hemoglobin in older populations. From a public health standpoint, our study highlights an urgent need to transition from purely clinical management of severe anemia to population-wide, proactive surveillance of mild and subclinical hemoglobin declines. Integrating hemoglobin screening into routine primary care and national healthy aging policies is a critical public health approach. Such interventions can effectively mitigate modifiable risk factors, preserve functional independence, reduce the systemic burden on healthcare infrastructure, and optimize overall survival in a rapidly aging global population.

## Data Availability

The raw data supporting the conclusions of this article will be made available by the authors, without undue reservation.
